# Senescent Cells in Cancer: Wanted or Unwanted Citizens

**DOI:** 10.3390/cells10123315

**Published:** 2021-11-26

**Authors:** Sven E. Niklander, Daniel W. Lambert, Keith D. Hunter

**Affiliations:** 1 Unidad de Patologia y Medicina Oral, Facultad de Odontologia, Universidad Andres Bello, Viña del Mar 2520000, Chile; 2 Unit of Oral and Maxillofacial Medicine and Pathology, School of Clinical Dentistry, University of Sheffield, Sheffield S10 2TA, UK; d.w.lambert@sheffield.ac.uk (D.W.L.); k.hunter@sheffield.ac.uk (K.D.H.); 3 Healthy Lifespan Institute, University of Sheffield, Sheffield S10 2TN, UK; 4 Oral Biology and Pathology, University of Pretoria, Pretoria 0028, South Africa

**Keywords:** senescence, SASP, cancer, carcinogenesis, senomorphics, senolytics

## Abstract

Over recent decades, the field of cellular senescence has attracted considerable attention due to its association with aging, the development of age-related diseases and cancer. Senescent cells are unable to proliferate, as the pathways responsible for initiating the cell cycle are irreversibly inhibited. Nevertheless, senescent cells accumulate in tissues and develop a pro-inflammatory secretome, known as the senescence-associated secretory phenotype (SASP), which can have serious deleterious effects if not properly regulated. There is increasing evidence suggesting senescent cells contribute to different stages of carcinogenesis in different anatomical sites, mainly due to the paracrine effects of the SASP. Thus, a new therapeutic field, known as senotherapeutics, has developed. In this review, we aim to discuss the molecular mechanisms underlying the senescence response and its relationship with cancer development, focusing on the link between senescence-related inflammation and cancer. We will also discuss different approaches to target senescent cells that might be of use for cancer treatment.

## 1. Introduction

Since the first description of senescence in the 1960s, when Hayflick and Moorhead found that human fibroblasts had a limited ability to proliferate in culture, senescence has been implicated in several different physiological processes including aging [1], wound healing [2], development [3] and tumour prevention [4]. The term cellular senescence refers to a cellular state characterized by permanent cell growth arrest in response to different stressors to avoid propagation of genetically damaged cells [5]. Despite the senescence response acting as an antitumour mechanism, there is compelling evidence that senescent cells can predispose to and promote tumourigenesis [6,7,8]. Senescent cells themselves do not transform into cancer, as they have lost growth capabilities, but the cells adjacent to them might be at risk. This is because senescent cells develop a secretory phenotype known as the senescence-associated secretory phenotype (SASP). The SASP is a pro-inflammatory plastic phenotype, in which the synthesis and/or release of more than 40 factors involved in intercellular signalling are increased [9]. Among those factors are different inflammatory molecules and extracellular vesicles able to promote tumour formation by acting in a paracrine manner [10,11,12]. The SASP from senescent fibroblasts has been shown to facilitate tumourigenesis of epithelial cancers, promoting the growth of cancerous cells of breast [6], skin [13] and prostate tumours [14] and enhancing peritoneal dissemination of gastric cancer [15]. Thus, different approaches have been utilized to develop drugs that target the SASP, whether by selectively eliminating senescent cells (senolytics) or by regulating its development (senomorphics) [16]. Here, we review the molecular mechanisms underlying the senescence response and its relationship with cancer development, focusing on the link between senescence-related inflammation and cancer. Finally, we discuss different approaches to target senescent cells and the possible therapeutic potential that this offers for cancer treatment.

## 2. Cellular Senescence

Replicative senescence is considered a potent tumour-suppressor mechanism [17,18,19] because it can stop cells with somatic mutations (considered as pre-cancerous cells) from dividing and acquiring further mutations that could enable replicative immortality and cancer development. When cells senesce, they remain metabolically active and fail to initiate DNA replication, despite mitogenic or oncogenic stimulation [13]. Most senescent cells become resistant to apoptosis, but it is not clear what determines if a cell senesces or undergoes apoptosis. This seems to be related to the cell type, and nature and intensity of the damage [20]. Senescent cells also change their gene expression profile [21] and secrete multiple pro-inflammatory molecules (cytokines, chemokines, growth factors, proteases) and extracellular vesicles; a phenotype recognized as the senescence associated secretory phenotype (SASP) [6,12] (Table 1). This is important as, despite senescence acting as an antitumour mechanism, it has been demonstrated that the SASP can promote tumour formation [22].

### 2.1. Inducers of Senescence

Senescence can be triggered by different inductors, including: replicative stress (replicative senescence) [23], DNA damage by chemotherapy and radiotherapy (therapy-induced senescence and/or radiation-induced senescence) [24,25], oxidative stress (oxidative stress-induced senescence) [26], oncogenic signalling (oncogene-induced senescence) [27] and other types of stresses causing DNA damage. All types of non-replicative senescence are usually grouped under the name of stress-induced premature senescence or premature senescence [28]. Despite the removal of the senescence trigger, cells will not return to a proliferative state, as senescence is considered an irreversible cell growth arrest [29].

Replicative senescence is the result of telomere shortening. After each cell division, the telomeres, DNA–protein structures that cap the end of linear chromosomes and protect them from degradation or fusion, shorten. This happens because DNA polymerase cannot replicate the end of a linear DNA molecule, losing 50–200 base pairs of telomeric DNA after each S phase [30]. Thus, telomeres function as a biological clock, limiting the cell proliferation capacity with every cell division (this is also known as the Hayflick limit) [31]. When telomeres erode and become dysfunctional, a DNA damage response (DDR) is triggered, because the DNA repair machineries recognize uncapped chromosome ends as a double strand break (DSB) inducing a senescence response (primarily through the p53 pathway), to avoid genomic instability [32]. Telomere shortening does not occur in cells that express telomerase, an enzyme with a catalytic component (telomerase reverse transcriptase or TERT) and an RNA template that allows the replenishment of telomeric DNA de novo [23]. Most somatic cells do not express telomerase or express low levels of it. Among the cells that normally express telomerase are embryonic stem cells, some adult stem cells and cancer cells [33].

Oncogene-induced senescence (OIS) is independent of telomeric length and can be induced either by the activation of oncogenes (such as RAS) [6,27] or by the loss of tumour suppressors (such as RB) [34]. It is commonly characterized by the activation of a DNA-damage response (DDR) induced by chromatinic accumulation of phosphorylated minor histone H2A variant (γH2AX) and p53-binding protein 1 [35]. Similar to OIS, therapy-induced senescence is also usually triggered by a DDR [36]. In oxidative stress-induced senescence, reactive oxygen species (ROS) activate p53 and/or p21^Waf1/Cip1^ to induce apoptosis or transient growth arrest [37]. If the stress is not resolved, cellular mechanisms will activate p16^Ink4a^ and/or pRB to induce an irreversible growth arrest [38].

### 2.2. Molecular Pathways Leading to Senescence

**Figure 1:**
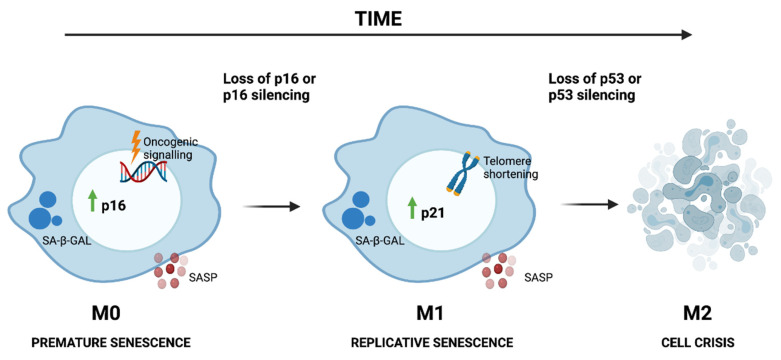
If cells are exposed to genotoxic stress, e.g., oncogene activation, senescence will be induced prematurely via p16INK4a activation, a step known as M0. If the cells bypass this mortality checkpoint because of p16INK4a silencing due to post-transcriptional modifications or mutations, senescence will be later induced by p53 and/or p21 activation due to telomere shortening (M1). If p53 is silenced or mutated, the cells can bypass M1 and proliferate further, until cell crisis (M2) is triggered due to chromosomal instabilities. For the cells to become immortal, they must express telomerase, which would help to bypass M2. Green arrow means increase. Image created with BioRender.

Whilst there are different inducers of senescence, senescence is established and maintained by two pathways, the p53 and/or p21^Waf1/Cip1^ and p16^Ink4a^ and/or pRB pathways [39,40] (Figure 1). The p16^Ink4a^ and/or pRB pathway is responsible for the early induction of senescence when cells are exposed to different stressors (such as oncogene activation), which is often termed mortality phase 0 (M0), first plateau or telomere-independent senescence, and happens usually after 10–20 population doublings (PDs). At this stage, senescent cells will express elevated levels of p16^Ink4a^, together with common senescence features, such as cell enlargement and increased SA-β-Gal activity [41]. However, if p16^Ink4a^ is silenced (by promoter methylation, mutation or deletion of the INK4a locus), cells will be able to continue dividing despite the DNA damage and will escape M0 [42]. Additionally, some cells arrested in M0 can undergo spontaneous mutations that will allow them to escape from the senescent state, which will give rise to clones without the aforementioned features [43]. In these cases, senescence will be later induced by telomere shortening. Telomere-initiated senescence is triggered mainly by the p53 and/or p21^Waf1/Cip1^ pathway [30] and is usually referred to as mortality phase 1 (M1) or second plateau. At M1, cells display the same senescent features as in M0 [41]. Cells that escape M1 due to loss of p53 expression can still divide, but just 20–30 PDs, until they reach mortality phase 2 (M2), characterized by extensive genomic instability, which leads to crisis and cell death (Figure 1). When the genes controlling M2 are inactivated, which often includes reactivation of telomerase, cells become immortal [20,44]. Irrespective of the pathway that triggered senescence, NF-κB seems to have a central role (as a downstream factor) in its induction. The activation of NF-κB induced by p53 and/or p21^Waf1/Cip1^ or p16^Ink4a^ and/or pRB pathways leads to the upregulation of IL-6 and IL-8, two cytokines that can induce and reinforce senescence in an autocrine or paracrine way [10,45].

### 2.3. Markers and Characteristics of Senescent Cells

In vitro, senescent cells acquire morphological alterations that differentiate them from non-senescent cells, such as enlargement, flattening, vacuolization and accumulation of stress granules. Despite this, there is no universal marker to identify senescent cells [46]. The most used marker to identify a senescent cell is the activity of senescence-associated β-galactosidase (SA-β-Gal), since it was first reported that β-galactosidase activity increases in senescent cells and not proliferating cells or quiescent cells [47]. SA-β-Gal corresponds to a lysosomal hydrolase enzyme that catalyses the hydrolysis of β-galactosidase into monosaccharides. When a specific substrate, such as X-GAL (the most commonly used substrate to assess SA-β-Gal activity) is combined with SA-β-Gal, it gets catalysed and dimerizes forming a blue precipitate, indicative of increased lysosomal activity [48]. Other commonly used markers are LaminB1, p16^Ink4a^ and p21^Waf1/Cip1^ expression. LaminB1 is a nuclear filament, the expression of which decreases during senescence and is reported to act as a senescence effector [49]. p16^Ink4a^ and p21^Waf1/Cip1^ are two common markers of senescence, as they are highly expressed in many senescent cells and are low or undetectable in normal tissue [10,40].

Some senescent cells also contain cytological markers of senescence-associated heterochromatin foci (SAHFs), which correspond to a reorganization of chromatin into foci, in which pro-proliferative genes have been silenced [5,33]. Accumulation of proteins associated with DNA damage, called senescence-associated DNA damage-foci (SDFs), can also be identified in some senescent cells, such as phosphorylation of the minor histone H2A variant (γH2AX) [50]. Nevertheless, the appearance of γH2AX foci is not a specific marker of senescence, as it can be observed as part of the DNA damage response independent of whether senescence is triggered or not, so it should be used together with other more specific markers [51].

None of these features should be used by themselves to identify senescent cells. A combination of these hallmarks should be used to reliably identify senescent cells, as single markers are inadequate [52]. In an attempt to standardize the identification of senescent cells, a recent guideline for the assessment of cellular senescence has been published [48]. For the assessment of senescence in vitro, the guidelines suggest the identification of at least three different traits: (i) the halt of cell cycle progression (by the assessment of p16^Ink4a^ and/or pRB, p53 and/or p21^Waf1/Cip1^, lack of Ki-67, among others), (ii) relevant structural changes (SA-β-Gal activity, SAHFs accumulation, loss of LaminB1, etc.), and (iii) an additional trait specific for the subtype of senescence being tested (e.g., DNA damage marker, specific SASP factors, ROS levels, etc.). The detection of senescent cells in ex vivo tissues is more challenging, as not all markers used for in vitro assessment can be applied. For example, most cells of the body are quiescent or terminally differentiated; thus, assessing lack of proliferation or DNA synthesis is not entirely appropriate. Additionally, SA-β-Gal activity, probably the most used marker for assessment of senescence in vitro, can only be detected in fresh tissues, which is a challenge as many studies use FFPE samples. The guideline recommends the immunohistochemical detection of at least three different markers within the same cells, which includes (i) a cell cycle marker such as of p16^Ink4a^ and/or pRB, p53 and/or p21^Waf1/Cip1^, (ii) increased lysosomal mass and content (SA-β-GAL, SBB or GL13), and (iii) relevant nuclear features (Lamin B1 loss, SAHFs, DDR proteins, DNA-SCARS) [48].

## 3. The Senescence-Associated Secretory Phenotype

Once cells senesce, they remain metabolically active and frequently adopt a pro-inflammatory state known as the SASP [33]. However, not every cell that senesces develops the SASP. Some cells can senesce without expressing a SASP [18]; why this happens remains unclear, but available evidence suggests that sole activation of p16^Ink4a^ without DNA damage is not sufficient for the development of the SASP [18]. When cells develop a SASP, different downstream effectors, such as NF-κB, p38MAPK, and C/EBPβ, which are transiently activated under normal conditions, become persistently activated [6,53,54] due to the induction of the p21^Waf1/Cip1^ and/or p53 and p16^Ink4a^ and/or pRB pathways. These lead to increased expression of different factors that characterize the SASP.

### 3.1. Characteristics and Functions

The SASP includes several families of soluble factors such as pro-inflammatory cytokines, growth factors, chemokines, proteases, protease regulators and small extracellular vesicles (sEVs) [12] (Table 1). These soluble factors and sEVs can induce transduction pathways in surrounding cells causing, for example, chronic inflammation [10]. The transcriptome and secretome of the SASP varies among cell types and with the stimuli that induced senescence (telomere shortening, oncogene activation, oxidative stress, etc.) [55,56], which reflects how heterogenous the SASP can be. Even in the same population of senescent cells, the expression of several senescence-associated genes varies [52]. Thus, the SASP is considered a plastic phenotype. Despite this, there is substantial overlap among SASPs, especially with pro-inflammatory cytokines such as IL-1α, IL-6 and IL-8, which are highly conserved among SASPs [57].

The SASP can be beneficial or detrimental, depending on different factors such as its composition, intensity, and the microenvironment [58]. Beneficial functions attributed to the SASP are immune surveillance, tumour suppressor activity, cell to cell communication, clearance of senescent cells, wound healing and paracrine induction and reinforcement of senescence [19,59]. Despite one of the most important functions of the SASP being its tumour suppressor activity exerted by the paracrine re-enforcement of senescence, pro-tumourigenic properties have been widely described [7,13,24,60].

### 3.2. Pathways Involved in the Regulation of the SASP

The regulation of senescence and the development of the SASP are fundamental for tissue homeostasis. If cells cannot senesce (because of mutations that help them bypass senescence, for example), this will predispose to cancer development. On the other hand, excessive accumulation of senescent cells, and their associated SASP, can lead to age-related diseases and cancer [29].

**Figure 2:**
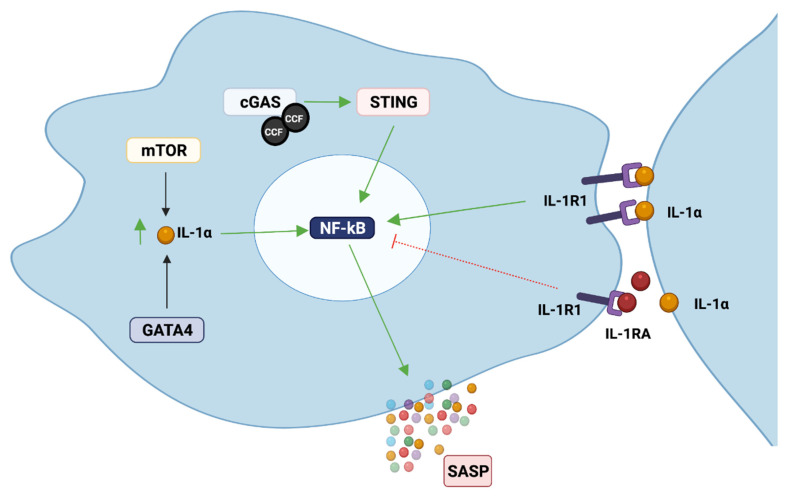
Examples of different pathways commonly implicated in the regulation of the SASP. The activation of these pathways converges in the activation of NF-κB, which is probably the most important pathway for the secretion of most of the inflammatory factors from the SASP. IL-1 inhibition with IL-1RA or neutralizing antibodies has shown to restrain the SASP by reducing NF-κB activation, highlighting the importance of IL-1/IL-1R1 signalling in this process. Green arrows mean stimulation, red inhibition. Image created with BioRender.

The SASP can be triggered by different factors, such as DNA damage [53], Toll-like receptors (TLR) [61], and cytoplasmic chromatin fragments (CCF) [62], and it is controlled at different levels: chromatin modification, transcription, secretion, mRNA stability and translation [63]. Different signalling pathways have been reported to be responsible for development and regulation of the SASP, which includes phosphoinositide-3-kinase (PI3K) [64], inflammasome [10], mammalian target of rapamycin (mTOR) [65,66,67], p38MAPK [53], STAT3 [68], GATA4 [69], and cGAS/STING [62,70,71], among others (Figure 2). These pathways mostly converge in the activation of two important transcription factors, NF-κB and CEBPβ [63], which are commonly activated in senescent cells. NF-κB and CEBPβ are important regulators of the key SASP factors IL-1α, IL-6 and IL-8, which in turn positively regulate NF-κB and CEBPβ activity, enhancing the development of the SASP [72,73].

IL-1α is probably the most important factor present in the SASP, as it is considered its major initiator and regulator. IL-1α expression has been reported to increase during senescence in different cell types, including oral keratinocytes, fibroblasts, endothelial cells [8,74,75,76], among others. In fibroblasts, cell surface IL-1α induces the secretion of IL-6 and IL-8, regardless of the senescence inducer [72], and IL-1α inhibition has been shown to moderate the pro-inflammatory component of the SASP [10]. It is suggested that IL-1α regulates the SASP via IL-1R1, as depleting cells of IRAK1 (a downstream kinase recruited after IL-1R1 activation) reduces IL-6 levels even after IL-1 stimulation. IL-1α depletion also reduces NF-κB activity, which is important for IL-6 and IL-8 secretion by senescent cells [72]. Similar results were reported in vascular smooth muscle cells (VSMC). IL-6 and IL-8 levels increased in senescent VSMC, and the blockage of IL-1α, but not IL-1β, reduced IL-6 and IL-8 in senescent VSMC [75]. In agreement with this, a recent transcriptome profile of IL-1R-depleted senescent cells showed that IL-1 signalling controls the NF-κB-dependent senescent secretome, and that disruption of the IL-1 signalling pathway uncouples the SASP from the senescent-associated cell cycle arrest [77]. This is of importance, as the SASP from IL-1α-depleted fibroblasts was shown to be less able to induce invasion of cancer breast cells compared to the SASP from wild type senescent fibroblasts, suggesting an important role of IL-1α for the oncogenic properties of the SASP [72]. Similar results have also been reported by others [8,77].

IL-1α is also important for the induction of senescence in a paracrine manner, probably by inducing p21 expression [65]. Knockdown of IL-1R1 or blockage of either IL-1α or IL-1β reduces paracrine senescence [10]. Therefore, IL-1α can increase the inflammatory component of the SASP by: (i) inducing paracrine senescence, thus increasing the number of cells contributing to the production of pro-inflammatory molecules, or (ii) by inducing NF-κB activation and production of inflammatory factors such as IL-6 and IL-8.

IL-1′s main inhibitor, IL-1RA, is also probably an important regulator of the senescence program and the SASP. We recently showed that IL-1RA levels decrease gradually during oral keratinocyte senescence, accompanied by an increase in IL-1α, IL-1β, IL-6 and IL-8 SASP factors. IL-1RA knockdown increased NF-κB activation and IL-6 and IL-8 production, which was associated with premature senescence [74].

Regulation of IL-1α to support the production of pro-inflammatory SASP factors has been reported to be controlled by the mTOR pathway [65]. mTOR inhibition in senescent fibroblasts decreased IL-1α production, reducing NF-κB activation and IL-6 and IL-8 secretion. Using polysome fractioning (which measures the fraction of an mRNA that is associated with polyribosomes), it was determined that mTOR inhibition decreased the translational efficiency of IL-1α mRNA [65]. Others have reported that mTOR also modulates the SASP by regulating the translation of MAPKAPK2 [67]. Persistent mTOR activation converts reversible cell cycle arrest (quiescence) into senescence [78], whereas mTOR inhibition results in quiescence [79]. Nevertheless, mTOR inhibition in already senescent cells does not reverse cellular senescence [65,67].

GATA4 is also an important regulator of IL-1α expression in senescent cells. In proliferative cells, GATA4 binds to p62 autophagy adaptor and is degraded by selective autophagy, whereas in senescent cells, GATA4 abundance increases as its interaction with p62 decreases. GATA4 stabilization leads to TRAF3IP2 (tumour necrosis factor receptor-associated factor interacting protein 2) and IL-1α production, resulting in NF-κB activation, which helps in the initiation and maintenance of the SASP [69]. GATA4 pathway activation is independent of p53 and p16^Ink4a^, but does depend on ATM and ATR activation, both important DDR kinases [69]. This agrees with a recent report of Malaquin et al. [80], where they showed that histone deacetylase inhibitors (HDACi) can trigger stable proliferation arrest and development of the SASP in the absence of DNA damage. HDACi-induced SASP requires the accumulation of ATM and MRN complexes on chromatin, revealing non-canonical DDR signalling underlying the SASP [81].

Recently, cyclic GMP-AMP synthase (cGAS), which stimulates the adaptor protein of stimulator of interferon genes (STING), was reported as an important inductor of senescence and the SASP [62,70,71]. cGAS is an important component of the innate immune system responsible for the detection of cytosolic DNA (e.g., from bacteria, viruses). When cGAS detects DNA in the cytosol, this triggers a type I interferon (IFN) response through the activation of STING [82]. During cellular senescence, the nuclear membrane weakens due to degradation of LaminB1 (an important structural protein of the nuclear membrane), and the chromatin undergoes reorganization and degradation. These changes facilitate the formation of nuclear blebs, which contain small fragments of chromatin that will be expelled from the nuclei into the cytoplasm, becoming cytoplasmic chromatin fragments (CCFs) [70]. These CCFs contain DNA and γH2AX and are recognized by cGAS, which binds and activates STING. STING activation will lead to the recruitment of TANK-binding kinase 1 (TBK1) and IkB kinase, which, through the activation of IFN regulatory factor 3 (IRF3) and NF-κB, will produce type I IFNs and different inflammatory cytokines [82]. CCF accumulation is controlled by two DNases, DNase2 and TREX1 (the enzymes responsible for its degradation), but both DNases are downregulated in senescent cells, which results in the accumulation of cytoplasmic DNA [71]. The cGAS-STING pathway can also be activated in the absence of cytosolic DNA. A recent study showed that exogenous IL-1β was able to activate IRF3 and TBK1 through STING phosphorylation, as cells lacking STING were unable to initiate such a response [80].

## 4. Cellular Senescence and Cancer

The accumulation of senescent cells has been associated with the development of aging and age-related diseases, including Alzheimer’s disease [83], Parkinson’s diseases [84], osteoarthritis [85], pulmonary diseases [86,87], cardiovascular diseases [88], obesity-induced metabolic dysfunction [89], macular degeneration [90] and cancer [9,15,33,58]. The association of aging with cancer is not new. Initially, this association was explained by the presence of age-related genetic mutations. Nevertheless, the current understanding of the tumour microenvironment (TME), in which its different components participate in the tumorigenesis process, and the significant differences between an aged stroma compared to a young stroma, led to the proposal that senescent cells could positively influence this process [91].

**Figure 3:**
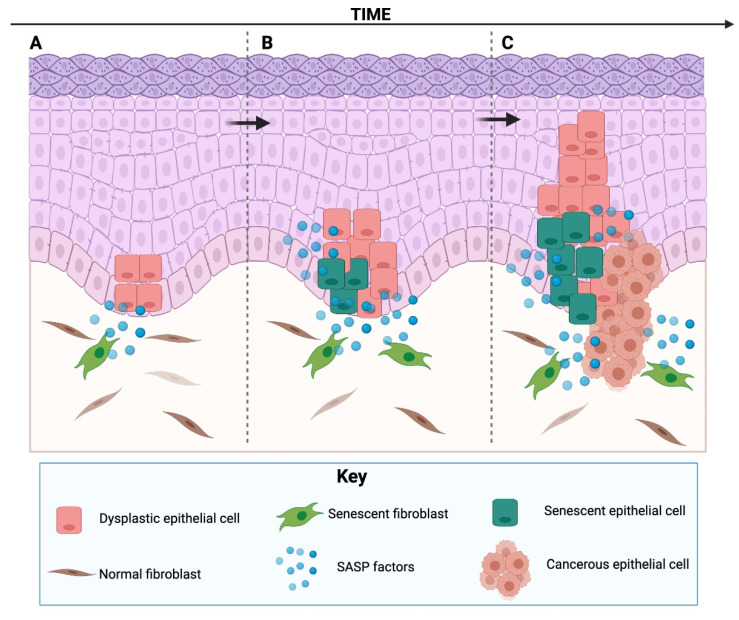
Proposal of how senescent cells could promote tumour development from a premalignant lesion and tumour progression. Adapted from Parkinson 2010 [92]. In epithelial dysplasia, dysplastic epithelial cells can benefit from SASP factors produced by stromal senescent cells, such as senescent fibroblasts (**A**). As time passes, SASP factors can promote proliferation of dysplastic cells and induce senescence of other stromal cells in a paracrine way. Some of the dysplastic cells will senesce as well, as they possess mutations that will trigger a DNA damage response to assure that they will not pass mutations to daughter cells, but some will escape senescence (**B**). If this is maintained over time (due to insufficient clearance of senescent cells or due to the expression of a de-regulated SASP), the remaining dysplastic keratinocytes can use these factors, proliferate further, achieve EMT and acquire an invasive phenotype, giving rise to a malignant tumour (**C**). Horizontal arrows mean progression over time. Image created with BioRender.

It is counterintuitive that one of the most potent existing antitumour mechanisms can also act the opposite way. Why would a mechanism designed to be beneficial for cells also be deleterious, promoting aging and related pathologies? To understand this, a theory based on antagonistic pleiotropy has been proposed [59]. According to the evolutionary theory, older individuals tend to be uncommon in natural populations, because natural mortality tends to be caused by extrinsic causes (infections, starvation, cold, etc.), preventing most individuals from reaching advanced ages. So, senescence as a tumour suppressor mechanism is designed to be effective only to ensure successful reproduction in young individuals, despite having possible deleterious effects in late life. Thus, these mechanisms, technically designed to prevent tumours in young organisms, can be detrimental in older organisms [59]. This is supported by the findings that senescent cells accumulate in older organisms (being extremely uncommon in young organisms) and are found at sites of age-related pathologies (Figure 3).

### 4.1. The SASP as a Tumour-Promoting Mechanism

There is substantial evidence that many of the factors from the SASP can create a microenvironment suitable for cancer development [9] (Figure 3). The SASP has been shown to induce cancer promotion, progression and metastasis in tumour cells from different origin, including: neural [93], breast [94,95], skin [22], gastric [15], oral [96], prostate [97] and ovary [98]. Most of the studies that have assessed the capabilities of senescent cells to promote cancer formation or metastasis have focused on senescent fibroblasts, as fibroblasts are the most abundant cells in the stroma, are responsible for the production of structural components of the stroma and the basal membrane, can secrete ECM components and ECM-degrading enzymes and can remain active for long periods of time [99]. The contribution of other senescent cells, such as keratinocytes [74], vascular cells [75], mesenchymal stem cells [94], has also been studied.

The SASP is regulated, at least in part, by wild type p53 [6], probably by regulating p38MAPK activation [53]. p38MAPK is reported to be important for the development of the SASP as, through NF-κB, it is able to initiate and maintain the SASP independently of canonical DDR signalling [53]. As TP53 is usually mutated in cancerous cells [100], this could lead to a stronger and uncontrolled SASP response in p53 mutated tumours, generating a chronic inflammatory tumour-promoting microenvironment. The strongest evidence of the pro-oncogenic properties of the SASP comes from xenograft studies. In mice, co-injection of senescent fibroblasts with premalignant epithelial cells promotes tumour formation [13] and co-injection of senescent fibroblasts with cancerous epithelial cells has been shown to stimulate tumourigenesis [13,101,102], which, in these studies, was attributed to the ability of senescent cells to produce VEGF, induce angiogenesis [101] and to secrete MMP3 [102].

IL-6 and IL-8 are the most robustly expressed cytokines of the SASP. These cytokines have been extensively studied as their presence as part of the SASP can promote tumour formation by: inducing epithelial-to-mesenchymal transition [6,103,104,105], stimulating angiogenesis and tumour growth [104,106], disrupting cell–cell communication [103], altering macrophage function, triggering innate immune responses and promoting epithelial migration and invasion [59]. Both IL-6 and IL-8 are induced by the activation of NF-κB, which is activated by the increased expression of IL-1α via IL-1R1 [45,72,107]. Different cancer models have shown IL-1 inhibition to moderate the expression of SASP factors in senescent fibroblasts [10] and to reduce the invasiveness of surrounding cancer cells [8,72].

### 4.2. Therapy Induced Senescence and Its Implications

**Figure 4:**
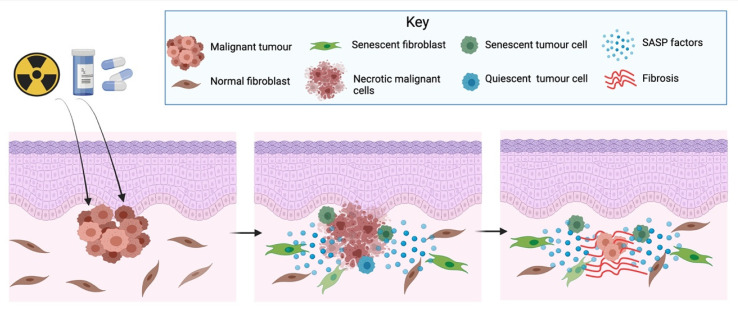
Radio- or chemotherapy will destroy most of the tumour cells, but some tumour cells will not die and will senesce or remain in a quiescent state. Additionally, some stromal cells, such as fibroblast, will also senesce. Clinically, it might appear that the tumour has disappeared, but the SASP from TIS cells can generate a pro-inflammatory TME that can stimulate quiescent tumour cells to re-enter the cell cycle, enabling tumour relapse. Horizontal arrows mean progression over time. Image created with BioRender.

Cancer treatment is multimodal. It includes surgery, radiotherapy, chemotherapy, immunotherapy, and hormone therapy, among other treatments. Some of these therapies, e.g., cisplatin, doxorubicin, and radiotherapy, are toxic to the cells and can induce a senescence response of not only cancer cells, but also of stromal cells [108,109,110], a phenomenon known as therapy-induced senescence (TIS) [92]. There is compelling evidence that TIS is not an in vitro observation, but also happens in vivo [111,112]. This is of importance, as stromal or cancer cells induced to senesce because of cancer therapy also develop a SASP, which can have various deleterious effects (Figure 4).

After cancer treatment, not all cancer cells will die or senesce, as some tumour cells develop the ability to escape cell death or TIS. It has been demonstrated that after the application of different chemotherapeutic agents (cisplatin, camptothecin, etoposide, paclitaxel and vindesine), up to 10% of the tumour cells enter a non-proliferative state reversible upon the overexpression of cyclin-dependent kinase Cdc2/Cdk1 [111]. Tumour cells can also become senescence-resistant, as shown by a study where some breast cancer cells developed senescence resistance to the chemotherapeutic agent adriamycin, probably by preventing the downregulation of Cdc-2 [113]. The underlying mechanisms predisposing for these processes are unknown, but it has been proposed that the SASP from TIS cells could help cancerous cells escape senescence and cell dormancy [112] by inducing EMT [114] (Figure 4). In addition, the SASP from TIS cells can also induce cell proliferation, promoting tumour relapse. Studies using bleomycin-induced senescent fibroblasts, cisplatin-induced senescent melanoma cells and irradiation-induced senescent fibroblast have shown these cells induce the growth of co-transplanted breast, melanoma, and lung cancer cells in in vivo animal models [102,115,116].

TIS has also been associated with other undesirable side-effects. Cisplatin treatment has been shown to induce renal senescence both in vitro and in vivo and to be associated with the development of renal interstitial fibrosis [117]. Radiation therapy induces senescence in the salivary gland stem/progenitor cell niche, which was associated with the development of radiation-induced hyposalivation. Removal of senescent cells by the senolytic drug ABT263 leads to increased stem cell removal and mitigated tissue degeneration in mice, preserving salivation [118]. Radiation-induced senescence is also reported to have considerable side effects in other organs, as has been involved in the development of pulmonary fibrosis [119], atherosclerosis [120] and cardiovascular damage [121]. Although senescence induction might be initially beneficial for cancer treatment, there is increasing evidence that excessive accumulation of senescent cells can have considerable side-effects, including cancer relapse. Therefore, there is a growing interest to develop pharmacological agents to target senescent as part of cancer treatment.

## 5. Targeting Senescent Cells in Cancer

Senescent cells are an important source of inflammatory molecules contributing to chronic inflammation, which predisposes to different diseases [112]. Therefore, it could be expected that their elimination has favourable effects. In fact, in mice, elimination of senescent cells has shown to increase mice lifespan, but more importantly, to reduce the burden of age-related deterioration and tumourigenesis [40,122]. Thus, attempts have been made to develop drugs that can target senescent cells (senotherapeutics), whether by interfering with their paracrine signalling (senomorphics) or by selectively killing them (senolytics) [123] (Table 2).

### 5.1. Senomorphic Agents

**Figure 5:**
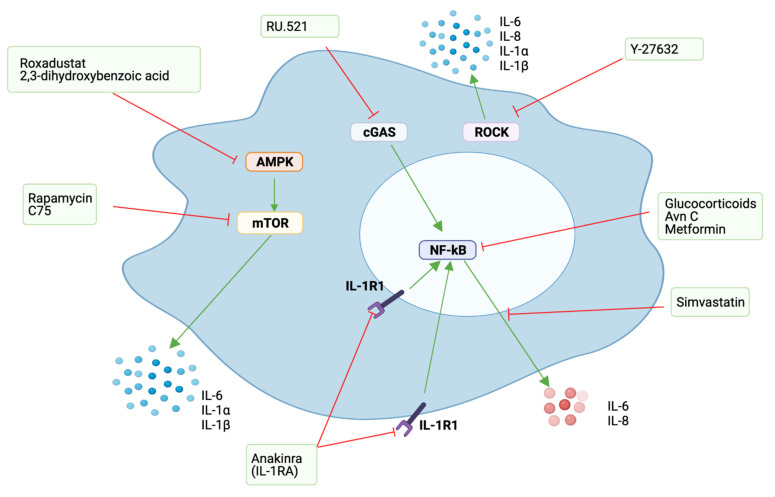
Different drugs with reported senomorphic effects and their different ways of action. Green arrows mean stimulation, red—inhibition. Avn C: Aventhramice C. Image created with BioRender.

In the context of tumourigenesis, senescence is still a beneficial response, preventing cells with oncogenic mutations from dividing, thus preventing cancer. Therefore, different approaches, using a variety of agents to target the SASP or key SASP factors, without compromising the senescent cell arrest, have been explored (Table 2, Figure 5). All these agents have shown different benefits by targeting the SASP, which suggests them as promising drugs for the treatment of some of the undesired effects of cellular senescence.

As most of the deleterious effects of the SASP are related to its capacity to induce chronic inflammation, one of the first approaches to regulate its expression was using glucocorticoids, as they are potent anti-inflammatory agents for different inflammatory conditions. Glucocorticoids are successful in reducing inflammatory SASP components associated with NF-κB signalling, including IL-6 [124]. Nevertheless, long-term use of glucocorticoids is associated with significant side-effects and drug resistance, which makes their use as senomorphics in clinical settings unlikely.

Other approaches targeting the NF-κB pathway have also been considered. Avenanthramice C (Avn C), a soluble phenolic compound extracted from oats, was successful in inhibiting the SASP by reducing the secretion of IL-6, IL-8 and TGF-β1 through the inhibition of the p38/NF-κB signalling pathway [125]. Metformin, an antidiabetic drug commonly use in clinical settings, has also been assessed as a SASP regulator, as it reduces NF-κB activity by preventing its translocation to the nucleus [126]. Different animal studies have shown metformin to have beneficial effects in age-related diseases, prolonging the health span and lifespan of mice [127] and inducing tumour reduction and remission [138,139]. IL-1α is an upstream regulator of the NF-κB pathway and is considered a master cytokine for the regulation of the SASP [77]. Thus, its inhibition has also been considered as an alternative to target the SASP. The major inhibitor of IL-1, IL-1RA, is also reported to regulate the SASP, as its knockdown is followed by an increase in IL-6 and IL-8 secretion and premature induction of senescence [74]. IL-1 inhibition, whether with neutralizing antibodies or with recombinant IL-1RA, have both been shown to regulate the SASP and to impair tumour progression [8,10,72,77].

Hypoxia is reported to suppress mTOR and geroconversion [78] and to reduce the levels of detrimental pro-inflammatory SASP factors [66]. Therefore, it has been suggested that low oxygen levels could decelerate premature senescence and extend lifespan [78]. In agreement with this, in vitro and in vivo experiments showed the hypoxia-mimetic compounds, Roxadustat and 2,3-dihydroxybenzoic acid, to suppress the SASP by restraining mTOR activity through AMPK activation. The suppression of the SASP was not attributed to elimination of senescent cells, as p16 and SA-βgal expression remained similar between the animals from the experimental and control groups [66]. Significantly, chemotherapy-treated aged mice treated with hypoxia-mimetic compounds had a significant improvement in muscular strength, compared to control mice. [66]. Fatty acid synthase inhibition (FASN) by the small molecule inhibitor of FASN, C75, has been also proposed as a potential alternative for SASP modulation by affecting mTOR activity. C75 reduced the expression of IL-1α IL-1β and IL-6 SASP factors and suppressed the secretion of small extracellular vesicles in mouse and human senescent cells. This was likely achieved by preventing the induction of senescence and by downregulating the expression of p53, an important SASP regulator [128]. Statins have also been reported for use in SASP modification, as simvastatin is able to reduce the production of IL-6 and IL-8 SASP factors [131].

cGAS knockdown has been shown to efficiently reduce the expression of SASP factors, which include IL-6, IL-8 and IL-1 [62,82]. The recent development of cGAS inhibitors, such as RU.521, which have been shown to reduce constitutive expression of interferon in a cGAS-selective way [140], could therefore have potential use as senomorphic agents. In fact, a recent study from our group showed RU.521 to partially moderate the SASP of dysplastic senescent oral keratinocytes by decreasing the secretion of IL-6 [74].

Recently, we proposed Rho kinase (ROCK) inhibition as a novel alternative for SASP regulation, as in in vitro experiments using senescent oral keratinocytes, Y-27632 (a commonly used ROCK inhibitor) was able to reduce the secreted levels of the signature SASP factors IL-1α, IL-1β, IL-6 and IL-8, without compromising the cell cycle arrest [129]. Similar results have been reported by others after [130].

### 5.2. Senolytic Agents

Senolytics are a relatively new group of drugs consisting usually of small molecule drugs able to selectively induce apoptosis of senescent cells (Table 2). Senescent cells have an active DDR and persist in tissues thanks to the activation of anti-apoptotic pathways, which includes: the Bcl-2/Bcl-xL anti-apoptotic pathway [135,137,141], the PI3K pathway [136,142] and the blockage of pro-apoptotic receptors [143]. Thus, in order to selectively eliminate senescent cells without affecting proliferating or quiescent cells, senolytics target these antiapoptotic pathways used as pro-survival mechanisms in senescent cells [144].

Some of these drugs have shown promising effects in mouse models. AT-406, a small molecule inhibitor of apoptosis inhibitor genes, induced apoptosis of senescent cells by regulating the expression of the anti-apoptotic proteins c-IAP2 and XIAP, decreasing the secretion of SASP factors. This created a pro-regenerative microenvironment, decreasing the progression of osteoarthritis in rats [122]. ABT263, a specific inhibitor of Bcl-2 and Bcl-xl, selectively killed senescent bone marrow hematopoietic cells and senescent muscle stem cells in mice. This led to a mitigation of total body-irradiation-induced premature aging of the hematopoietic system, and rejuvenated aged hematopoietic stem cells and muscle stem cells in normally aged mice [132]. In another study, ABT263 was also shown to improve salivary gland hypofunction in irradiated mice, by eliminating salivary gland senescent stem/progenitor cells [118]. Navitoclax, another inhibitor of Bcl-2 family member proteins, was found to eliminate senescent human umbilical vein epithelial cells, senescent human lung fibroblasts and senescent murine embryonic fibroblasts, but not senescent human preadipocytes [133], suggesting a cell-specific way of action. This is of clinical importance as atissue-specificity of some of these drugs is suggested, limiting clinical applications. Other agents, A1331852 and A1155463, which induce apoptosis of senescent cells by selectively targeting Bcl-xl, have also been developed [135].

The combined used of dasatinib (a Bcr-Abl tyrosine kinase inhibitor) and quercetin (a kinase inhibitor) in an in vivo mouse model was shown to reduce the population of senescent cells, to increase cardiac function and exercise capacity, and to extend mice health span [134]. A recent clinical trial in humans showed promising results by using the same combination of drugs (dasatinib and quercetin) for the treatment of idiopathic pulmonary fibrosis (an age-related disease associated with the accumulation of senescent cells), improving the patient’s physical dysfunction [86].

Chimeric antigen receptor (CAR) T cells have been also tested as possible senolytic agents. CART cells targeting the urokinase-type plasminogen activator receptor (uPAR), a cell surface protein induced to express during senescence, efficiently eliminate senescent cells in vitro and in vivo [145]. More importantly, CART cells targeting uPAR extended the survival of mice with adenocarcinoma treated with a combination of senescence-inducing drugs and restored homeostasis in mice with liver fibrosis [145].

### 5.3. Future Perspectives

Reversal of cellular aging by reprogramming aged cells into induced pluripotent stem cells (iPCSs) is a new approach that has been explored to ameliorate age-associated symptoms [146]. Lapasset et al. (2011) were able to generate iPSC from senescent fibroblasts by transfecting a six-factor gene cocktail, which included OCT4, SOX2, KLF4, c-MYC, NANOG and LIN28. All iPSC generated from senescent fibroblasts restored their proliferative state and were able to differentiate into the three embryonic lineages [147]. Similar results have also been shown in an in vivo progeroid mouse model, where the reprogrammed mice showed amelioration in age-associated symptoms, lifespan prolongation and improvement in tissue homeostasis [148]. Cellular reprograming is an exciting development and might lead to new therapeutic strategies for the treatment of age-related diseases. Nevertheless, care must be taken, as in vivo reprogramming has been associated with tumour formation [149].

## 6. Concluding Remarks

Cellular senescence is a complex cellular response crucial for maintaining homeostasis. Senescent cells accumulate during aging, and although they have lost their proliferative capabilities (and, thus, cannot form tumours themselves), their accumulation in tissues is associated with age-related diseases and cancer, which is explained by the development of the SASP. During the last decade, there has been a significant development in the study of senescence, the SASP and their role in cancer development, generating convincing evidence to support a pro-oncogenic role of probably the most potent antitumour response of our body. This led to the development of a new field in drug research known as senotherapeutics. The use of senomorphics and senolytic agents for the treatment of age-related diseases and cancer holds promise. Nevertheless, most of these drugs were not initially designed for these purposes; thus, they can have significant off-target effects, limiting their clinical use. Additionally, some of them, such as the senolytic agent Navitoclax, have cell-specific susceptibility, suggesting utility for specific conditions. Therefore, more animal models are needed to further test these agents to grant their use in large clinical trials.

## Figures and Tables

**Table 1:** List of commonly reported SASP factors in senescent cells.

SASP Factors	
Inflammatory cytokines	IL-6, IL-7, IL-1⍺, IL-1β, IL-11, IL-13, IL-15
Chemokines	CXCL8 (IL-8), CXCL-1, -2, -3, -19, MCP-1, -2, -3, -4, MIP-1a, MIP-3a, HCC-4, Eotaxin-3, I-309
Growth factors and regulators	VEGF, EGF, HGF, KGF, Amphiregulin, Epiregulin, Heregulin, Angiogenin, SCF, SDF-1, IGFBP-1, -2, -3, -4, -5, -6, -7, IGFBP-rP1, -2
Protease and regulators	MMP-1, -3, -10, -12, -13, -14, TIMP-2, PAI-1, -2, uPa, tPa, Cathepsin B
Others	PGE2, Nitric oxide, ROS, ICAM-1, -3, OPG, sTNFRI, TRAIL-R3, Fas, uPAR, SGP130, EGF-3, Leptin, Osteoprotegerin, SCF, Extracellular vesicles, MIF, GM-CSF, G-CSF

IL: Interleukin; CXCL: CXC motif chemokine ligand; MCP: Monocyte chemoattractant protein; MIP: Macrophage inflammatory protein; LEC: Liver-expressed chemokine; CCL1: Chemokine C-C motif ligand 1; VEGF: Vascular endothelial growth factor; EGF: Epithelial growth factor; HGF: Hepatocyte growth factor; KGF: Keratinocyte growth factor; SCF: Stem cell factor; SDF-1: Stromal cell-derived factor 1; IGFBP: Insulin-like growth factor binding protein; IGFBPrP: Insulin-like growth factor binding protein-associated protein; MMP: Matrix metalloproteinase; TIMP-2: Tissue inhibitor of metalloproteinases 2; PAI: Plasminogen activator inhibitor; uPA: Urokinase-type plasminogen activator; tPA: Tissue plasminogen activator; PGE2: Prostaglandin E2; ROS: Reactive oxygen species; ICAM: Intercellular adhesion molecule; OPG: Osteoprotegerin; sTNFRI: Soluble tumour necrosis factor receptor I; TRAIL-R3: TRAIL receptor 3; uPAR: Urokinase plasminogen activator surface receptor; SGP130: Soluble GP130 protein; MIF: Macrophage migration inhibitory factor; GM-CSF: Granulocyte-macrophage colony-stimulating factor; G-CSF: Granulocyte colony-stimulating factor.

**Table 2:** Examples of different compounds that have been proposed as senomorphic or senolityc agents.

**SENOMORPHICS**		
**Agent**	**Mode of Action**	**Refs**
Glucocorticoids	Inhibits arachinoid acid metabolism	[124]
Aventhramice C (Avn C)	Inhibits NF-κB signalling	[125]
Metformin	Inhibits NF-κB signalling	[126,127]
Interleukin 1 receptor antagonist (Anakinra)	IL-1 inhibitor	[8,10,72,77]
Rapamycin	mTOR inhibitor	[65,67]
Roxadustat	Hypoxia-mimetic, targets mTOR	[66]
2,3-dihydroxybenzoic acid	Hypoxia-mimetic, HIF prolyl hydroxylase inhibitor	[66]
C75	Fatty acid synthase (FASN) inhibitor	[128]
RU.521	cGAS inhibitor	[74]
Y-27632	Rho kinase (ROCK) inhibitor	[129,130]
Simvastatin	Targets IL-6, IL-8 and MCP1	[131]
**SENOLYTICS**		
**Agent**	**Mode of Action**	**Refs**
AT-406	Regulates anti-apoptotic proteins c-IAP2 and XIAP	[122]
ABT263	Bcl-2 and Bcl-xl inhibitor	[132]
Navitoclax	Bcl-2 and Bcl-xl inhibitor	[133]
Dasatinib	Tyrosine kinase inhibitor	[86,134]
Quercetin	Flavonoid	[86,134]
Fisetin	Flavonoidº	[135,136]
A1331852	Selective Bcl-xl inhibitor	[135]
A1155463	Selective Bcl-xl inhibitor	[135]
Geldanamycin	HSP90 inhibitor	[137]
17-AAG (tanespimycin)	HSP90 inhibitor	[137]

## Data Availability

Not applicable.
